# The Roles of MicroRNAs (miRNAs) in Avian Response to Viral Infection and Pathogenesis of Avian Immunosuppressive Diseases

**DOI:** 10.3390/ijms20215454

**Published:** 2019-11-01

**Authors:** Linyi Zhou, Shijun J. Zheng

**Affiliations:** 1 Key Laboratory of Animal Epidemiology of the Ministry of Agriculture, Beijing 100193, China; zlyi123321@126.com; 2 College of Veterinary Medicine, China Agricultural University, Beijing 100193, China

**Keywords:** miRNA, immunosuppressive diseases, chickens, host response

## Abstract

MicroRNAs (miRNAs) are a class of non-coding small RNAs that play important roles in the regulation of various biological processes including cell development and differentiation, apoptosis, tumorigenesis, immunoregulation and viral infections. Avian immunosuppressive diseases refer to those avian diseases caused by pathogens that target and damage the immune organs or cells of the host, increasing susceptibility to other microbial infections and the risk of failure in subsequent vaccination against other diseases. As such, once a disease with an immunosuppressive feature occurs in flocks, it would be difficult for the stakeholders to have an optimal economic income. Infectious bursal disease (IBD), avian leukemia (AL), Marek’s disease (MD), chicken infectious anemia (CIA), reticuloendotheliosis (RE) and avian reovirus infection are on the top list of commonly-seen avian diseases with a feature of immunosuppression, posing an unmeasurable threat to the poultry industry across the globe. Understanding the pathogenesis of avian immunosuppressive disease is the basis for disease prevention and control. miRNAs have been shown to be involved in host response to pathogenic infections in chickens, including regulation of immunity, tumorigenesis, cell proliferation and viral replication. Here we summarize current knowledge on the roles of miRNAs in avian response to viral infection and pathogenesis of avian immunosuppressive diseases, in particular, MD, AL, IBD and RE.

## 1. Introduction

MicroRNAs (miRNAs) are a class of non-coding single-stranded RNA molecules encoded by endogenous genes of approximately 20 to 24 nucleotide (nt) in length [1]. Since miRNAs were first discovered in *Caenorhabditis elegans*, more and more miRNAs have been identified in humans, animals, plants and viruses [2,3,4]. With the advance in miRNA research, the way miRNAs are produced and function is well clarified [5]. The maturity of miRNA includes the following stages: First, the non-coding region of DNA is transcribed by RNA polymerase I to form a precursor transcript (primary miRNA, pri-miRNA) with a hat structure (7MGpppG) and a poly(A) tail [6]. The pri-miRNA is then subjected to cleavage by RNase III nuclease Drosha and cofactor Pasha to generate a miRNA precursor (pre-miRNA) with a hairpin structure of approximately 70–90 nt [7]. Pre-miRNA is exported to the cytoplasm by RanGTP-dependent exportin 5 [8]. Subsequently, the pre-miRNA is hydrolyzed and digested into a double-stranded miRNA of 20 to 24 nt in length, which consists of a guide strand miRNA and an incompletely complementary passenger strand miRNA* [9]. The duplex is unwounded by helicase and miRNA guide strand then combined with the RNA-induced silencing complex (RISC) to recognize the target gene. The passenger strand miRNA* is usually rapidly degraded in the cytoplasm [10]. The mature miRNA recognizes the target gene through the 3′UTR of the target gene mRNA. When the mature miRNA is fully complementary to the target mRNA, the target mRNA will be degraded. However, if their sequences are not fully paired, the translation of the target mRNA will be inhibited and the integrity of the mRNA will not be affected [10]. miRNAs have the broadest range of gene regulation and regulate a variety of biological processes, including early embryo development, cell proliferation and differentiation, apoptosis and death, viral infection and tumorigenesis [11,12,13].

Avian immunosuppressive disease refers to a series of infectious diseases that result in damage or loss of function to the immune system of the poultry after infection. Viruses that cause immunosuppression in chickens include Marek’s disease virus (MDV), reticuloendotheliosis virus (REV), avian leukosis virus (ALV), infectious bursal disease virus (IBDV), chicken infectious anemia virus (CIAV) and avian reovirus (ARV) [14]. The infection of chickens with these viruses not only causes death, but also increases susceptibility to other microbial infections and the risk of failure in subsequent vaccination against other diseases, leading to serious economic losses [14]. It has been reported that miRNAs are involved in a number of regulatory activities in the response of chickens with avian immunosuppressive diseases, including tumorigenesis, immunosuppression, cell proliferation and viral replication [13,15]. In this review, the roles of miRNAs in the pathogenesis and control of different avian immunosuppressive diseases will be discussed, which may provide some clues to further understandings of pathogenesis of avian immunosuppressive diseases.

## 2. miRNA in Marek’s Disease (MD)

### 2.1. Marek’s Disease Virus (MDV)-Encoded miRNA

Marek’s disease is an important immunosuppressive and tumorigenic disease characterized by T lymphoid tissue cell proliferation triggered by infection with MDV [16]. According to different antigenic structures between different strains, MDV can be divided into three serotypes [17]. Among them, serum type 1 (MDV-1 or Gallid herpesvirus 2) is pathogenic, while serum type 2 (MDV-2 or Gallid herpesvirus 3) and serum type 3 (MDV-3 or Melagarid herpesvirus, MeHV-1) are apathogenic. The MDV-1-encoded miRNA was first identified among the three MDV serotypes, and eight virus-encoded miRNAs were found in chicken embryo fibroblast (CEF) infected with MDV-1 virulent strain RB-1B [18]. Subsequently, more virus-encoded miRNAs were found in the MDV-1 induced lymphoma cell line MSB-1, and currently, 14 precursor sequences in the MDV-1 genome that produce 26 mature miRNAs have been recorded [19]. These precursor sequences can be divided into three clusters according to their positions on the MDV-1 genome, which are Meq-cluster (including miR-M2, miR-M3, miR-M4, miR-M5, miR-M9 and miR-M12), Mid-cluster (including miR-M1, miR-M11 and miR-M31) and latent-related transcribed region (LAT)-cluster (including miR-M6, miR-M7, miR-M8, miR-M10 and miR-M13) [20]. Meq-cluster and Mid-cluster are located upstream and downstream of the oncogenic gene meq of MDV-1, respectively, and the LAT-cluster is located in the latent-related transcribed region (LAT) [21]. Meq-cluster and Mid-cluster are involved in tumorigenesis, and LAT-cluster in latent infection.

After the MDV-1-encoded miRNAs were discovered, MDV-2- and MDV-3-encoded miRNAs were identified. MDV-2 encodes 18 miRNA precursors (yielding 36 mature miRNA molecules) [22], and MDV-3 encodes 17 miRNA precursors (yielding 28 mature miRNA molecules) [23]. Alignment of miRNAs encoded by different serotypes of MDV revealed that miRNAs encoded by MDV-1, MDV-2 and MDV-3 are highly conserved in the viral genome although they have no homology in the gene sequence [22,24], suggesting that genome replication during the evolution of MDV-encoded miRNAs plays an important role [24]. However, the detailed information is still lacking in terms of the relation of MDV serotypes to MDV-encoded miRNAs.

### 2.2. The Role of MDV1-Encoded miRNAs in MD

Due to the tumorigenicity of MDV-1, MDV-1 encoded miRNAs are the most widely and intensively studied among the three serotypes. Interestingly, it was found that deficiency of the whole three miRNA clusters in MDV-1 virus did not affect the in vivo and in vitro replication of MDV but abolished the tumorigenicity of MDV, and the loss of carcinogenicity appears to be mainly due to the absence of miR-M4 in Meq-cluster [25]. However, deletions of MDV1-miR-M4 from very virulent (vv) MDV strain GX0101 decreased rather than abolished its oncogenicity [26]. MDV1-miR-M4 is the most abundant MDV-1-encoded miRNA in tumors, and its expression takes up to 72% of all MDV-1-encoded miRNAs [27]. MDV-1-miR-M4 is a homolog of cellular miR-155. miR-155 has been identified as tumorigenic miRNA and is closely related to tumorigenesis [28]. This suggests that MDV1-miR-M4 is an important tumorigenic molecule encoded by the virus and may play varied roles in the pathogenesis of different virulent strains of MDV-1.

It seems that MDV1-miR-M4 promotes MDV-mediated tumors primarily through two aspects. On the one hand, MDV1-miR-M4 targets tumor-associated cellular genes to promote tumorigenesis (Table 1). For example, both MDV1-miR-M4-5P and gga-miR-155 target six tumor-associated cellular genes to effectively promote tumorigenesis [29], and MDV1-miR-M4-5p activates oncogene c-Myc by targeting latent transforming growth factor beta binding protein 1 (*LTBP1*) and inhibiting transforming growth factor beta (TGF-β) signaling pathway, thereby promoting MDV-induced tumorigenesis [30]. It was also found that MDV1-miR-M4-5p promotes proliferation of chicken embryo fibroblast (CEF) and DF-1 cells by targeting heterogeneous nuclear ribonucleoprotein A/B (*hnRNPAB*) [31]. On the other hand, MDV1-miR-M4-5p is involved in regulating the expression of MDV-1 viral unique long gene 28 (*UL28*) and unique long gene 32(*UL32*) to promote the maintenance of MDV-1 latency [29]. However, a recent study indicates that MDV1-miR-M4, although essential for MDV-induced tumorigenesis, is not required for maintaining the proliferation of transformed phenotypes and transformed cells [32]. In addition to promoting tumorigenesis, MDV1-miR-M4 was found to be involved in the regulation of innate immune molecules to promote viral replication (Table 1). Furthermore, MDV1-miR-M4 up-regulates adenosine deaminase RNA specific 1 (*ADAR1*) expression by indirectly targeting suppressor of cytokine signaling 1 (*SOCS1*) mRNA, further down-regulates type I interferon (IFN) production and promotes viral replication [33]. Toll-like receptor 3 (TLR3), a pattern recognition receptor, inhibits the replication of MDV-1 by binding to ligands to initiate activation of the TLR3-mediated signaling pathway [34]. It was found that MDV1-miR-M4-5p was involved in the regulation of TLR3 expression [35], suggesting that MDV suppresses TLR3-mediated immune signaling via the regulation of TLR3 expression by miR-M4-5p. In addition to miR-M4, other miRNAs in Meq-cluster have also been confirmed to play a role in promoting tumorigenesis (Table 1). Infection of specific-pathogen-free (SPF) chickens with miR-M2-, miR-M3-, miR-M5-, miR-M9- and miR-M12-deleted MDV-1 strains reduced tumorigenesis to varying degrees [36]. MDV1-miR-M3 inhibits Cisplatin-induced apoptosis by down-regulating the expression of SMAD family member 2 (Smad2) protein, creating an environment conducive to viral latency and tumorigenesis [37]. MDV1-miR-M5-3P was found to downregulate viral infected cell protein 22 (*ICP22*) mRNA expression during latency, and ICP22 has been shown to be required for the lytic replication of MDV-1 [38]. However, little information is available regarding the mechanisms by which miRNAs in Meq-cluster, such as MDV-1-miR-M2, -miR-M9 and -miR-M12, promote tumorigenesis.

Mid-cluster and LAT-cluster miRNAs are also involved in MDV-1 mediated tumorigenesis (Table 1). It was reported that the deletion of Mid-cluster of miRNAs from MDV has no effect on viral replication and viral gene expression [39], suggesting that Mid-cluster of miRNAs is not required for the replication of MDV-1. The exact role of Mid-cluster miRNAs in the pathogenesis of MDV infection needs to be investigated. In contrast to the parental MDV strain, infection of chickens with viral strains deficient of miR-M31 had reduced mortality and tumor incidence, but the pathogenicity and tumorigenicity of miR-M1- or miR-M11-deficient strains increased, suggesting that miR-M31-3p and miR-M11-5p function as oncogenes or tumor suppressor genes, respectively, in MD tumorigenesis [39]. There are few reports available on the function of LAT-cluster miRNAs although LAT-cluster miRNAs are considered to be involved in the establishment and maintenance of MDV-1 latent infection [27]. It was found that LAT-cluster’s miR-M7-5p targets and regulates the expressions of infected cell protein 4 (ICP4) and infected cell protein 27 (ICP27), which are involved in the regulation of MDV latent infection [40].

### 2.3. The Role of Cellular miRNAs in MD

Infection with MDV-1 causes a series of complicated responses in the host, including immunosuppression, neurological symptoms and tumor formation [16]. The exploration of the interaction between the virus and its host is conducive to the understandings of the pathogenesis of viral infection. With the development of sequencing technology, a large number of differentially expressed miRNAs of a host with MDV-1 infection were identified. The profile of differential expressions of host miRNAs in MDV-infected CEFs was first examined in avian cells, showing that 125,463 high-quality sequences were completely aligned to the chicken genome (Ensembl 12/06), and 63 new miRNAs were found [41]. Global miRNA expression profiles of seven different MDV transformed cell lines were detected by microarray analysis, and it was found that miR-150, miR-223 and miR-155 were down-regulated in different cell lines, suggesting that they are closely related to MDV-1 induced tumors [42].

**Figure 1:**
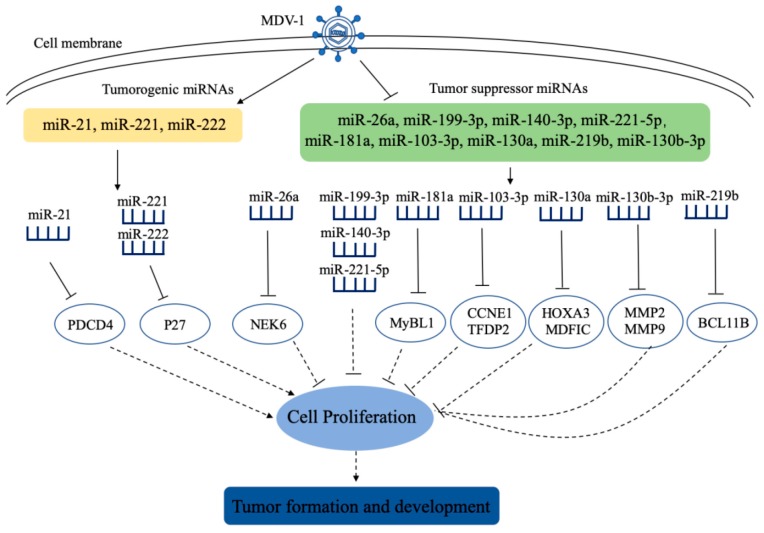
Schematic diagram of the roles of cellular miRNAs in the pathogenesis of Marek’s disease virus (MDV)-1 infection. After MDV-1 infects cells, the expressions of some cellular miRNAs are upregulated or downregulated, and they participate in MDV-1 induced tumors by targeting related genes. PDCD4: programmed cell death 4; NEK6: Never In Mitosis Gene A (NIMA)-related kinase 6; MYBL1: MYB proto-oncogene like 1; CCNE1: cyclin E1; TFDP2: transcription factor Dp-2; HOXA3: Homeobox A3; MDFIC: myogenic differentiation (MyoD) family inhibitor domain containing; MMP2: matrix metallopeptidase 2; MMP9: matrix metallopeptidase 9; BCL11B: B-cell chronic lymphocytic /lymphoma 11B.

With further research in the pathogenesis of MDV infection, a number of host miRNAs involved in regulating MDV-induced tumors have been discovered (Figure 1). Some tumorigenic miRNAs are up-regulated after viral infection, and they promote tumorigenesis by targeting genes that inhibit tumorigenesis [43,44]. For example, up-regulated miR-221 and miR-222 promote tumorigenesis by targeting the cell cycle regulatory molecule p27 (Kip1) [43]. Viral oncoprotein Meq binds to transcriptional factor AP-1 to upregulate gga-miR-21 expression, targeting chicken programmed cell death 4 (PDCD4) and promoting tumor cell growth and apoptosis escape [44]. In addition, some host miRNAs that inhibit tumorigenesis are down-regulated after viral infection, thereby promoting cell proliferation and tumor formation [45,46,47,48,49,50,51,52]. Gga-miR-26a was down-regulated in MDV-infected chicken spleen and targeted the cell proliferation-related molecule Never In Mitosis Gene A (NIMA)-related kinase 6 (*NEK6*) to inhibit the cell proliferation [46]. Gga-miR-199-3p, gga-miR-140-3p, and gga-miR-221-5p were down-regulated in MD lymphoma and inhibited the MSB-1 proliferation [47]. Gga-miR-181a inhibited MDV-transformed lymphocyte proliferation by targeting proto-oncogene like 1 (*MYBL1*) and was downregulated after MDV-1 infection [48]. gga-miR-130a was downregulated in an MDV-infected spleen, and inhibited proliferation and migration of MSB-1 by targeting homeobox A3 (*HOXA3*) and myogenic differentiation (MyoD) family inhibitor domain containing (*MDFIC*) [49]. Gga-miR-219b downregulated in tumorous spleen inhibited MSB-1 proliferation, migration and invasion by targeting B-cell chronic lymphoma 11B (*BCL11B*) [50]. The expression of gga-miR-103-3p decreased in tissues infected with MDV, and gga-miR-103-3p targeted cyclin E1 (*CCNE1*) and transcription factor Dp-2 (*TFDP2*) to inhibit cell migration [51]. MDV-1 infection inhibited gga-miR-130b-3p expression by promoting methylation level of upstream region of the Gga-miR-130b-3p gene, and gga-miR-130b-3p inhibited cell proliferation, migration and invasion by targeting matrix metallopeptidase 2 (*MMP2*) and matrix metallopeptidase 9 (*MMP9*) [52]. Although the role and mechanisms of cellular miRNAs in MDV-1 mediated tumors have been well studied, cellular miRNAs involved in other responses of host to MDV-1 infection have rarely been studied, such as immunosuppression and neurological symptoms. It will be intriguing to orient future researches to the effects of cellular miRNAs on immune response as well as other responses of host to MDV-1 infection.

## 3. miRNA in Avian Leukemia (AL)

### 3.1. Avian Leukosis Virus (ALV)-Encoded miRNA

Avian leukemia is a neoplastic disease caused by ALV [53]. According to the host range and antigenicity of ALV, ALV can be divided into 10 subgroups. Among them, avian exogenous ALV includes five subgroups of ALV A~D and ALV-J. Avian endogenous ALV includes the E subgroup [53]. Recently, a newly discovered K subgroup has been reported [54]. Among the known ALV subgroups of infected chickens, ALV-J is the most pathogenic and infectious. When ALV-J infects chickens, it will cause tumor formation in multiple organs, such as spleen, liver, kidney and bursa [53]. Most of the research on miRNAs for ALV is focused on ALV-J.

Unlike DNA viruses, virus-encoded miRNAs are rarely found in RNA viruses. Deep sequencing of ALV-J-transformed turkey macrophage cell lines IAH30 revealed an exogenous virus-specific region (E element or XSR) encoded miRNA of ALV-J, which was named as E (XSR) miRNA [55]. This miRNA of 148 nt is produced by a classical miRNA biogenesis pathway and has been shown to be active in RISC and is processed into functional mature miRNA in DF-1 cells [55]. Functional studies of E (XSR) miRNAs indicate that this miRNA is dispensable for ALV-J-induced tumors, but it contributes to the carcinogenicity of certain chicken genetic lines [56]. More effort will be required to investigate the exact mechanism underlying the biogenesis of virus-encoded miRNAs and its evolutionary and physiological relevance.

### 3.2. The Role of Cellular miRNAs in AL

Examination of miRNA expression profiles in the host after ALV-J infection is an initial step to explore the pathogenesis of ALV-J. In recent years, a large number of differentially-expressed miRNAs in different tissues and cells infected with ALV-J have been sequenced and analyzed. It was reported in 2012 that miRNA expressions were detected in the liver of 10-week-old chickens with or without ALV-J infection by miRNA microarray analysis [57]. The data from this study show that 12 miRNAs were differentially expressed in the liver of chickens in infected groups compared with the uninfected controls. Out of 12 miRNAs, seven were upregulated by ALV-J infection and possibly related to carcinogenicity, and five were down-regulated by ALV-J infection and likely related to tumor suppression [57]. Another study shows that four differentially expressed miRNAs, gga-miR-221, gga-miR-193a, gga-miR-193b and gga-miR-125b were detected in the hepatic tumor tissues of chickens 238 days after ALV-J infection, and the differentially expressed miRNAs are involved in some tumorigenesis-related signaling pathways, such as the MAPK signaling pathway and the Wnt signaling pathway [58]. Up to now, the differential expressions of host miRNAs have been examined in varied primary cell cultures, cell lines and chicken tissues with ALV infection. ALV transformed B-cell line DT40 showed changes in miRNA expression in relation to the naive B cells [59]. miRNA profile of ALV-J-infected CEF cells revealed some differentially expressed miRNAs associated with cell proliferation and Wnt/β-catenin pathway [60]. Sequencing of exosomal RNAs from CEF cells co-infected with ALV-J and REV identified a total of 54 (23 upregulated and 31 downregulated) miRNA genes by comparing with that from CEF cells infected with ALV-J [61]. Transcriptome analysis of ALV-J infected chicken primary monocyte-derived macrophages (MDMs) revealed some differentially expressed miRNAs associated with host antiviral responses [62] and it was reported that 167 miRNAs were differentially expressed in the spleen of ALV-J-infected chickens compared to that of uninfected controls [63]. With no doubt, this information will provide a valuable clue to uncover the role of miRNAs in host response to ALV infection.

**Figure 2:**
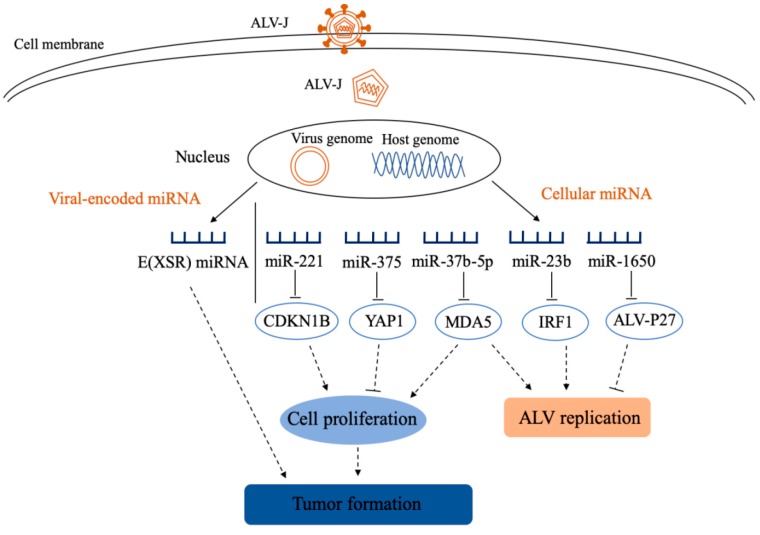
Schematic diagram of the roles of miRNAs in the pathogenesis of avian leukosis virus (ALV)-J infection. After ALV-J infection, viral-encoded miRNA and host miRNAs are involved in tumorigenesis and viral replication. CDKN1B: cyclin dependent kinase inhibitor 1B; YAP1: yes-associated protein 1; MDA5: melanoma differentiation associated gene 5; IRF1: interferon regulatory factor 1.

After ALV-J infection, cellular miRNAs are involved in the pathogenesis of ALV-J by affecting ALV-J-mediated tumor and viral replication (Figure 2). Cellular miRNAs affect ALV-J replication by targeting immune-related molecules or viral genes. It was reported that gga-miR-1650 decreases ALV-J replication by binding to the 5′UTR of ALV-J genome, and overexpression of gga-miR-1650 downregulates Gag protein expression [64]. Gga-miR-23b was found to be more expressed in the spleen of ALV-J-infected chickens than that of controls, and may play an important role in ALV-J replication by targeting interferon regulatory factor 1 (*IRF1*) [63]. Similarly, miR-34b-5p was significantly up-regulated in chicken spleens, targeting melanoma differentiation associated gene 5 (*MDA5*) to accelerate proliferation and migration of ALV-J-infected cells and promotes ALV-J replication via inhibiting MDA5 signaling pathway [65]. In addition to affecting viral replication, cellular miRNAs are also involved in ALV-J-induced tumors by targeting cell proliferation-associated genes (Figure 2). A report demonstrates a significant down-regulation of gga-miR-375 expression in chicken liver 20 days after infection with ALV-J, and overexpression of gga-miR-375 significantly inhibited proliferation of DF-1 cells by directly targeting yes-associated protein 1 (*YAP1*) [66]. Furthermore, gga-miR-221 promotes cell proliferation and cell cycle progression by targeting cyclin-dependent kinase inhibitor 1B (*CDKN1B*), which is beneficial to ALV-J-induced tumors [67]. These cellular miRNAs seem to be exploited by ALV for its own benefit. The exact mechanism of how ALV regulates these “beneficial miRNAs” needs to be clarified.

## 4. miRNA in infectious bursal disease (IBD)

### 4.1. The Role of miRNA in the Pathogenesis of Infectious Bursal Disease Virus (IBDV)

**Figure 3:**
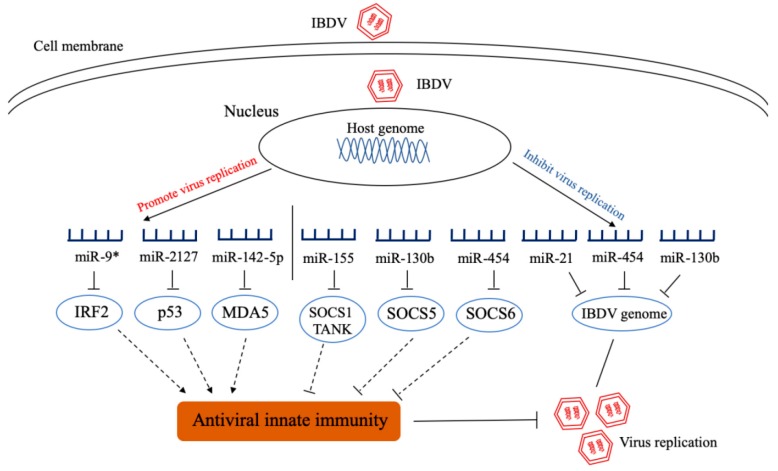
Schematic diagram of the roles of cellular miRNAs in host response to infectious bursal disease virus (IBDV) infection and viral replication. After IBDV infection, some cellular miRNAs were differentially expressed. These miRNAs promote or inhibit virus replication by directly targeting the genome of IBDV or molecules that regulate innate immunity. IRF2: interferon regulatory factor 2; MDA5: melanoma differentiation associated gene 5; SOCS1: suppressor of cytokine signaling 1; TANK: TRAF family member associated NF-κB activator; SOCS5: suppressor of cytokine signaling 5; SOCS6: suppressor of cytokine signaling 6.

Infectious bursal disease, also called Gumboro disease, is an acute, highly contagious and immunosuppressive poultry disease caused by IBDV. The consequent immunosuppression of IBD increases susceptibility to other microbial infections and the risk of failure in subsequent vaccination against other diseases [68]. This disease still threatens the poultry industry worldwide, and in particular the frequent emergence of very virulence or variant IBDV strains in vaccinated flocks causes severe economic losses to stakeholders. Currently, there are no reports available regarding IBDV-encoded miRNAs. Our data from deep-sequencing of IBDV-infected DF-1 cells demonstrate that 369 host miRNAs were significantly upregulated and 169 downregulated in DF-1 cells with IBDV infection, and these differentially expressed miRNAs, as predicated by Kyoto Encyclopedia of Genes and Genomes (KEEG) and Gene Ontology (GO), are putatively involved in several immune-related signaling pathways, including JAK-STAT signaling pathway, Toll-like receptor-mediated signaling pathway, RIG-I-like receptor (RLR)-mediated signaling pathway and cytokine-cytokine receptor signaling pathway [69]. Recent studies have revealed the roles of differentially expressed miRNAs in IBDV infection and replication (Figure 3). It was found that gga-miR-9* inhibits the production of antiviral IFN by targeting interferon regulatory factor 2 (*IRF2*), promoting IBDV replication [70], and gga-miR-2127 down-regulates p53 translation and attenuates chp53-mediated innate immune responses to promote viral replication [71]. By directly targeting the 3′ untranslated region of *chMDA5*, gga-miR-142-5p attenuates interferon regulatory factor 7 (IRF7) signaling and promotes IBDV replication [72]. All these three miRNAs (gga-miR-9*, gga-miR-2127, and gga-miR-142-5p) favor IBDV replication. On the contrary, some miRNAs inhibit viral replication by directly targeting viral genome and/or promoting innate immunity (Figure 3). We found that gga-miR-454 inhibits viral replication by directly targeting IBDV genome B and suppressor of cytokine signaling 6 (*SOCS6*) [73], gga-miR-155 increases type I interferon expression by targeting suppressor of cytokine signaling 1 (*SOCS1*) and TRAF family member associated NF-κB activator (*TANK*), thereby inhibiting viral replication [74] and gga-miR-130b enhances IFN-β expression by targeting viral genomes and the cellular suppressor of cytokine signaling 5 (*SOCS5*), thereby inhibiting viral replication [69]. In addition, gga-miR-21 directly targets the virus *vp1* to inhibit viral replication by inhibiting viral protein translation rather than regulating host response [75]. Currently, it is still not clear how IBDV infection alters the expressions of cellular miRNAs that have completely opposite effects on IBDV replication.

### 4.2. The Role of miRNA in the Control of IBDV

Using the mechanism by which miRNAs function, miRNAs that target important genes of viruses could be constructed into recombinant viral vectors that might be used for the control of viruses. It was reported that the miRNAs expressed by recombinant miRNA expression vectors targeting *vp1* and *vp2* genes in the IBDV genome could effectively inhibit the replication of IBDV when transfected into DF1 cells [76,77]. In addition to viral genes, some miRNAs of host genes that have been shown to play important roles in IBDV infection are also designed to inhibit IBDV replication. As chicken heat shock protein 90 (cHsp90) serves as a functional component of cell receptor for IBDV infection, the vector-expressed anti-cHsp90α miRNA could effectively inhibit IBDV replication [78]. More efforts will be required to employ miRNAs for the control of IBDV infection.

## 5. miRNA in Reticuloendotheliosis (RE)

Reticuloendotheliosis is an infectious tumor-causing disease of poultry caused by REV [79]. REV has two types of replication, complete replication (REV-A strain) and incomplete replication (REV-T Strain). The REV-T strain is derived from the large deletion of the gag-pol gene and the env gene of the REV-A strain [80]. REV-T infection mainly leads to acute reticulocyte tumors in chickens. While REV-A strain can cause various symptoms such as reticuloendothelial hyperplasia, immunosuppression and lymphosarcoma [79].

It was reported that REV-T upregulates gga-miR-155 expression in CEF cells after infection, while gga-miR-155 promotes cell survival by targeting jumonji and AT-rich interaction domain containing 2 (*JARID2*), a cell cycle regulator, and facilitates viral replication [81]. It was found that a total of 88 differentially expressed miRNAs were identified in the bursa of Fabricius of SPF chickens 21 and 28 days after REV-A infection, and some of these miRNAs were found to be involved in processes such as apoptosis, tumorigenesis and cell proliferation [82]. For example, miRNAs gga-miR-18a-5p, gga-miR-155 and gga-miR-222b-5p, anti-apoptotic molecules, were found to be down-regulated 21 days after infection [82]. However, the role of these differentially expressed miRNAs in REV-A pathogenesis remains unclear.

## 6. Conclusions

Lines of evidence indicate that miRNAs play important roles in the pathogenesis of the avian immunosuppressive disease. Most of the information is gleaned from the studies on the pathogenesis of MDV, ALV or IBDV infections. Some miRNAs (miRNA-21, miRNA-221, miRNA-222, miRNA-26a, miRNA-199-3p, miRNA-140-3p, miRNA-221-5p miRNA-181a, miRNA-103-3p, miRNA-130a, miRNA-130b-3p, miRNA-219b, miRNA-375 and miRNA-34b-5p) are involved in regulation of virus-mediated tumor formation, which might provide some clues to the development of tumor therapy. However, the roles of miRNAs in host response to avian viral infection are still far from elucidation and need to be further investigated. In addition to MDV, ALV and IBDV, the commonly-seen viruses that cause immunosuppression in chickens include REV, CIAV and ARV. Currently, there is little information available regarding miRNAs related to REV CIAV and ARV. Further investigation into the role of miRNAs in these avian immunosuppressive viruses is conducive to elucidation of the pathogenesis of viral infections and the prevention and control of avian diseases.

## Figures and Tables

**Table 1:** The role of Marek’s disease virus (MDV)-1 encoded miRNAs summarized in this review.

Cluster	miRNA	Target Gene	Characteristics/Functions	References
Meq-cluster	MDV1-miR-M9	/	Promote tumorigenesis	[35]
	MDV1-miR-M5	*ICP22*	Promote tumorigenesis	[35,37]
	MDV1-miR-M12	/	Promote tumorigenesis	[35]
	MDV1-miR-M3	*Smad2*	Inhibit cisplatin-induced apoptosis; Promote tumorigenesis	[35,36]
	MDV1-miR-M2	/	Promote tumorigenesis	[35]
	MDV1-miR-M4-5p	*GPM6B, RREB1, c-Myb, MAP3K7IP2, PU.1, LTBP1, C/EBP, hnRNPAB*	Mimics cellular mir-155; Promote cell proliferation and tumorigenesis	[24,25,28,29,30,31]
		*SOCS1*	Inhibit interferon production and promote viral replication	[32]
	MDV1-miR-M4-3p	*UL28, UL32*	Prevent lytic replication and promote latency	[28]
Mid-cluster	MDV1-miR-M11	/	Inhibit tumorigenesis	[38]
	MDV1-miR-M31	/	Promote tumorigenesis	[38]
	MDV1-miR-M1	*ICP22*	Inhibit tumorigenesis	[37,38]
LAT-cluster	MDV1-miR-M7	*ICP4, ICP27*	Establish and/or maintain latency	[39]

Abbreviation: MDV: Marek’s disease virus; LAT: latency-associated transcript; ICP4: infected cell protein 4; ICP27: infected cell protein 27; ICP22: infected cell protein 22; Smad 2: SMAD family member 2; GPM6B: glycoprotein M6B; RREB1: ras responsive element binding protein 1; c-Myb: MYB proto-oncogene; MAP3K7IP2: TGF-beta activated kinase 1 (MAP3K7) binding protein 2; PU.1: Spi-1 proto-oncogene; LTBP1: latent transforming growth factor beta binding protein 1; C/EBP: CCAAT/enhancer binding protein; hnRNPAB: heterogeneous nuclear ribonucleoprotein A/B; SOCS1: suppressor of cytokine signaling 1; UL28: unique long gene 28; UL32: unique long gene 32.
